# Kiwi: a tool for integration and visualization of network topology and gene-set analysis

**DOI:** 10.1186/s12859-014-0408-9

**Published:** 2014-12-11

**Authors:** Leif Väremo, Francesco Gatto, Jens Nielsen

**Affiliations:** Department of Chemical and Biological Engineering, Chalmers University of Technology, Gothenburg, 412 96 Sweden

**Keywords:** Gene-set analysis, Transcriptomics, Network analysis, Visualization tool

## Abstract

**Background:**

The analysis of high-throughput data in biology is aided by integrative approaches such as gene-set analysis. Gene-sets can represent well-defined biological entities (e.g. metabolites) that interact in networks (e.g. metabolic networks), to exert their function within the cell. Data interpretation can benefit from incorporating the underlying network, but there are currently no optimal methods that link gene-set analysis and network structures.

**Results:**

Here we present Kiwi, a new tool that processes output data from gene-set analysis and integrates them with a network structure such that the inherent connectivity between gene-sets, i.e. not simply the gene overlap, becomes apparent. In two case studies, we demonstrate that standard gene-set analysis points at metabolites regulated in the interrogated condition. Nevertheless, only the integration of the interactions between these metabolites provides an extra layer of information that highlights how they are tightly connected in the metabolic network.

**Conclusions:**

Kiwi is a tool that enhances interpretability of high-throughput data. It allows the users not only to discover a list of significant entities or processes as in gene-set analysis, but also to visualize whether these entities or processes are isolated or connected by means of their biological interaction. Kiwi is available as a Python package at http://www.sysbio.se/kiwi and an online tool in the BioMet Toolbox at http://www.biomet-toolbox.org.

**Electronic supplementary material:**

The online version of this article (doi:10.1186/s12859-014-0408-9) contains supplementary material, which is available to authorized users.

## Background

Gene-set analysis (GSA) is a widely used category of bioinformatics methods and there are many available tools that perform GSA [1,2]. In GSA, genes known to contribute to a certain function, or share a relevant biological feature, are collected into sets. If these gene-sets are enriched by transcriptome or other high-throughput data, GSA directly highlights the most prominent among these sets, and thereby the underlying functions that are implicated by the data [2]. Networks stand at the basis of complex biological systems [3] and in many cases gene-sets represent elements that are connected, not simply because of gene overlap, but rather to exert a coordinated function through their interactions (the gene-set interaction network). Examples of elements that can be used as gene-sets and where an interaction network can be defined include: transcription factors in a gene regulatory network [4]; the hierarchical network of Gene Ontology terms [5]; and metabolite gene-sets in a metabolic network [6]. In particular the last example provides a very useful case since metabolite gene-sets (genes that are associated to reactions in which the metabolite takes part in) are connected through reaction pathways, but will usually not share any common genes (unless they participate in the same reaction). Thus, when several metabolite gene-sets in a pathway are significant their important biological connection will be lost, unless the gene-set interaction network is taken into account.

With this in mind, interpretation and visualization of the results from a GSA currently suffers from several limitations. Typically, the results are presented as a list of the most significant gene-sets, or visualized in a heatmap where gene-sets are clustered according to either the pattern of significance across several conditions or their direction of regulation. In both cases, the biologically relevant connections between gene-sets, defined by their interaction network, are ignored. Multiple connected significant gene-sets will likely represent an important biological process, but with the current visualization approaches these connections are lost and are tedious to elucidate manually.

On the other hand, it is not unusual to see GSA results presented as networks, with nodes representing the most significant gene-sets [1,7-9]. However, in these cases edges between nodes simply represent gene overlap. This can help to reduce the bias from redundant gene-sets by clustering gene-sets with overlapping gene content together. Nevertheless, a network visualization approach where the edges represent gene-set interactions is advantageous in the context of biological interpretation. Indeed, different tools can be used to visualize data on gene-set interaction networks [10-14], although some of them are not specifically made for that purpose. Unfortunately, these tools suffer from one or several of the following drawbacks:

- The tool is not made specifically to handle GSA data, which requires the user to tweak the input (e.g. common identifiers and color-coding scheme) in the best way possible to fit the framework of that tool.
- The tool is only made for a specific type of network (e.g. KEGG pathways or GO-terms), constraining the user to only one single gene-set type.
- The tool is not effectively reducing the network to highlight the significant results, but instead simply overlaying the data on the original, and potentially huge, gene-set interaction network.

Here we address the current limitations by developing a new network-based visualization approach and implement it in the software tool Kiwi. Contrary to other available tools, Kiwi explicitly embraces the paradigm that gene-sets can be biological entities that interact and it therefore aims at visualizing GSA results in the context of the gene-set interaction network in such way that the biological connections between all significant gene-sets become apparent. This is done by taking into account both the directionality and significance of the gene-sets and by removing non-interesting gene-sets from the visualized network. Further on, Kiwi is made as general as possible, in the sense that it accepts input from any GSA tool and any gene-set interaction network defined by the user. Finally, since the biological measurements behind the data are made at the gene-level, Kiwi enables the user to go from the visualization network of significant gene-sets back to the gene-level data, in order to detect driver genes behind the regulated biological elements that the gene-sets represent.

## Implementation

### Input data

The input to Kiwi is at minimum the gene-set interaction network and a table of p-values for the gene-sets, which can be collected from the output of any GSA tool. Apart from this, it is recommended to also supply the gene members of the gene-sets as well as the gene-level statistics (e.g. p-values and fold-changes) that were used as input to the GSA. Full details and required format for the input files can be found in the online Kiwi reference manual.

### Processing

**Figure 1:**
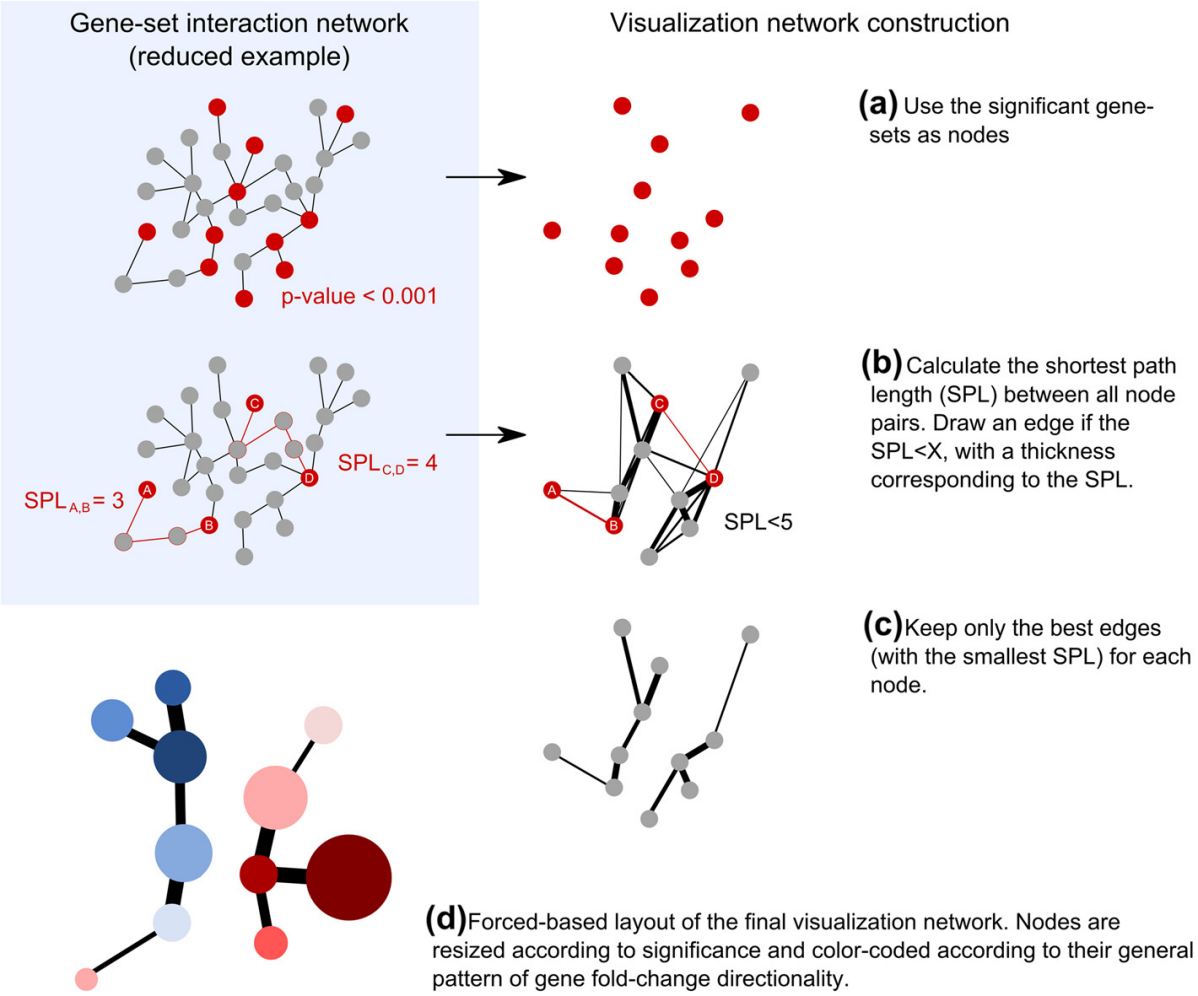
**Overview of the Kiwi workflow. (a)** Significant gene-sets are selected based on a user-set cutoff and used as nodes in the visualization network. **(b)** The shortest path length (SPL) between all node pairs in the gene-set interaction network is calculated. In the example, the SPL between node A and B is 3, and between node C and D is 4. If the SPL between two nodes is below a user-set cutoff (5 in the example), an edge is drawn between those nodes, with a thickness corresponding to the SPL (an SPL = 1 will generate the thickest edge). In the example, the edges between nodes A and B, and C and D, respectively, are marked in red, corresponding to the SPLs shown in the gene-set interaction network. **(c)** To avoid a cluttered network with too many edges, only the best edges (with lowest SPL) are kept for each node. Note that a node may still have multiple edges of different thickness if, for example, a thinner edge is the best one of a neighbouring node. (This step is optional.) **(d)** Finally, the visualization network is drawn using a forced-based layout. Nodes are resized according to the gene-set significance and color-coded in order to reflect the general direction of change of the gene-set.

An outline of the network visualization process performed by Kiwi is shown in Figure 1. First, non-significant gene-sets are filtered out according to a user-set cutoff. The remaining gene-sets are used as nodes in a new visualization network. In this visualization network the edges between gene-sets should reflect how closely they interact. The shortest path length (SPL) measures the shortest distance between two gene-sets and is a property of the network that indicates whether the two gene-sets are interacting directly or indirectly via a certain number of intermediates. Hence, the SPL between all pair of nodes in the gene-set interaction network is calculated. If the SPL between two gene-set nodes is below a user-set cutoff an edge is drawn between those nodes, with an edge thickness relative to the SPL. The SPL cutoff can be seen as a measure of the relatedness of two gene-sets in the gene-set interaction network, and it controls at what distance these gene-sets should not any longer be considered biologically connected. For each node, only the edge or edges with the lowest SPL are kept, so that each node is connected only to its closest nodes of those present in the visualization network. Finally, the visualization network is drawn using a force-based layout. Nodes are resized to reflect the gene-set significance and color-coded to capture the general direction of change of the genes in the set (refer to the online documentation for further details).

### Output

Kiwi produces two figures: a network and a heatmap. The network presents an uncluttered view where the most important features are highlighted. The node sizes and color-codes are adjusted according to the gene-set significance and general direction of change. The heatmap serves as a complement to the network by displaying the gene-level statistics for each gene-set in the network. The rows (gene-sets) and columns (genes) are hierarchically clustered, which enables the identification of (i) gene-sets with similar gene content and (ii) the significant genes that are driving the observed changes. Both figures can be fine-tuned by the user through several parameters and the network can also be saved in graphML format and imported into Cytoscape for further customization.

### Case studies

To illustrate the advantages of Kiwi, we use two case studies. The first one is based on a differential gene expression dataset from lung adenocarcinoma vs. normal lung tissue [15]. Metabolites from a human genome-scale metabolic model [16] were used as gene-sets and the GSA was carried out using the Bioconductor R-package piano [1].

For the second case study we used gene expression data from a study on Kras conditional activation in mouse xenograft tumors [17]. Metabolites from a mouse genome-scale metabolic model, derived from the human genome-scale metabolic model used in case study 1, using gene homology as described in [18], were used as gene-sets. The GSA was carried out using the Bioconductor R-package piano.

**Figure 2:**
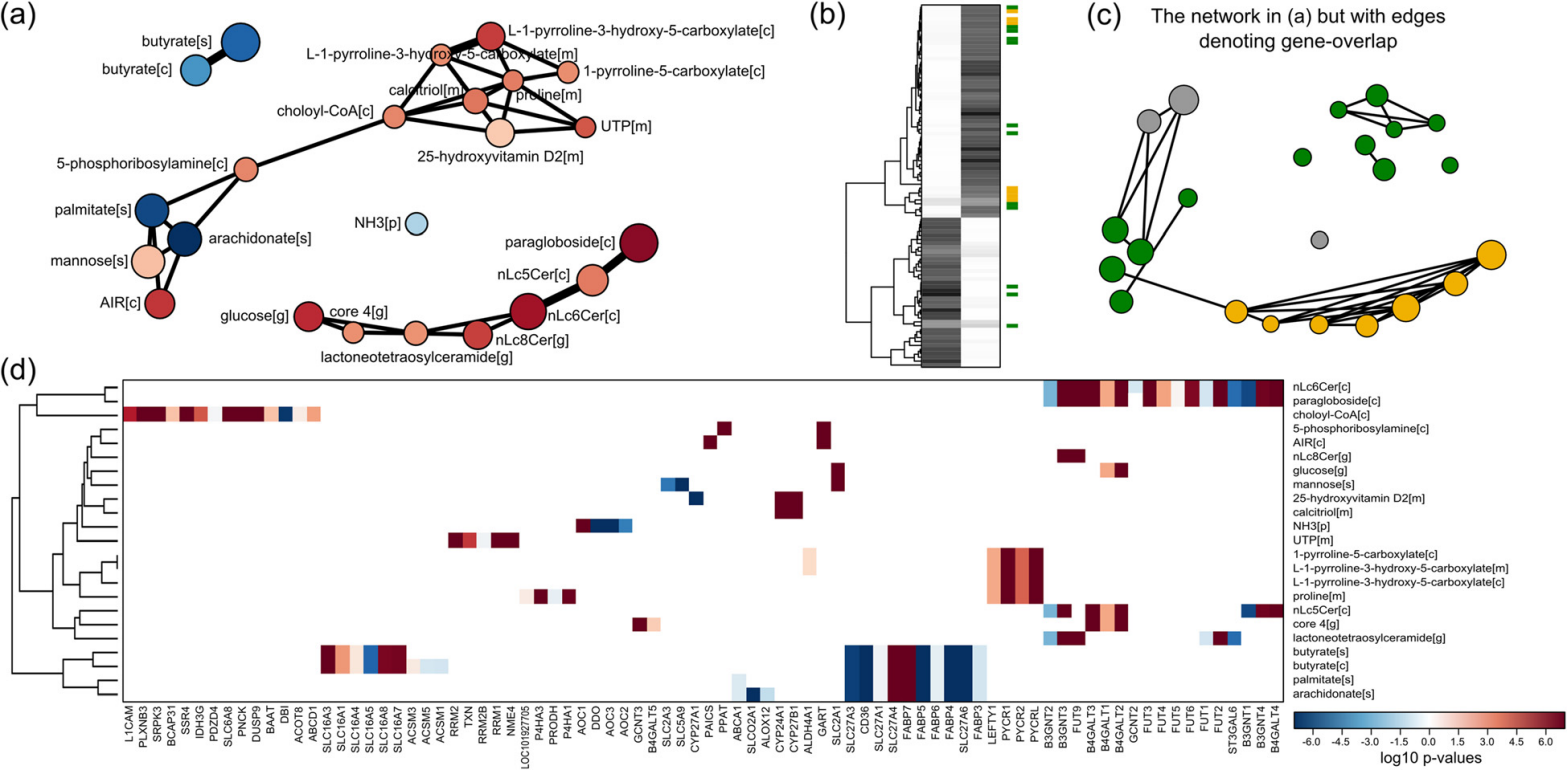
**The output from Kiwi in case study 1, metabolic changes upon transformation to lung adenocarcinoma. (a)** The Kiwi network shows the significant gene-sets and their general direction of change. Up-regulation: red, down-regulation: blue. Two metabolically connected pathways emerge, a pattern that is not easily detected in **(b)** a traditional heatmap or **(c)** a gene-overlap network. Green and orange indicate gene-sets of the two pathways, respectively. **(d)** The Kiwi heatmap displays the individual gene-level statistics for each significant gene-set. It also clusters similar gene-sets based on gene overlap in order to identify driver genes or potential bias.

**Figure 3:**
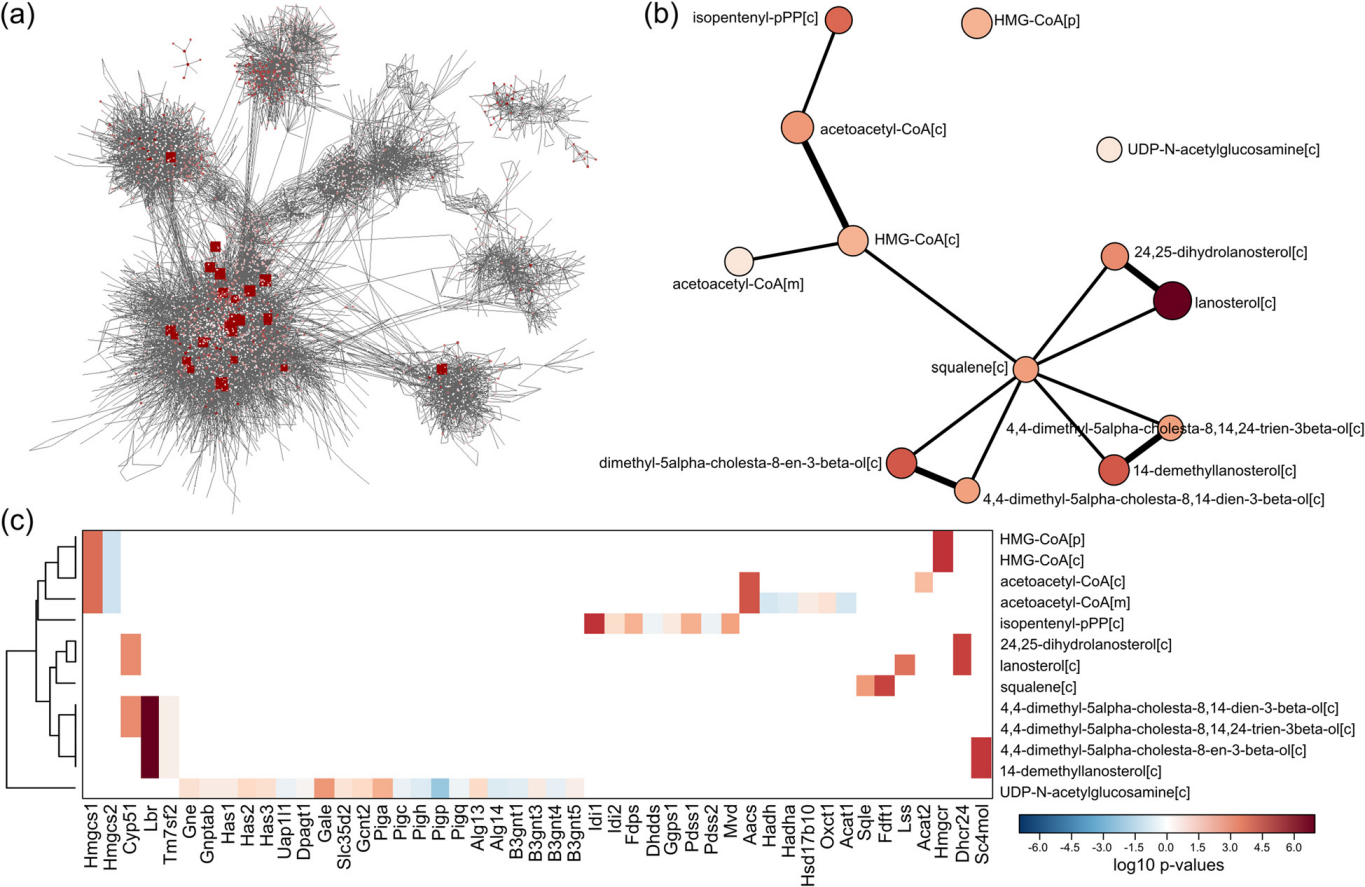
**The output from Kiwi in case study 2, metabolic changes upon activation of Kras in mouse tumors. (a)** The significant gene-sets overlayed on the metabolite-metabolite network (the full gene-set interaction network). As the network is too big and the gene-sets are too spread around the network it is not possible, in a simple way, to draw any biological conclusions about the data. **(b)** Kiwi effectively reduces the gene-set interaction network and pulls out the significant gene-sets, while maintaining their biological relatedness given in the original network. Here, Kiwi outputs a network showing the steroid biosynthetic pathway, in line with the original study. **(c)** The heatmap for the gene-sets in the Kiwi network shows that isopentenyl-pPP, 14-demethyllanosterol, squalene, and lanosterol are not overlapping in terms of gene members, yet they are connected in the Kiwi network.

Kiwi version 0.2.8 was used for both case studies. The heatmaps and network plots shown in Figure 2a,d and Figure 3b,c are the direct output from Kiwi, however, to provide as clear of a figure as possible, the node labels in the networks have been manually shifted. The data and scripts for running these case studies are available as Additional file 1.

## Results and discussion

In order to show the advantages, in terms of biological interpretation, of using Kiwi to visualize GSA results in the context of a gene-set interaction network, we performed two case studies. In both cases we used a genome-scale metabolic model to define a metabolite-metabolite network (connecting metabolites if they are substrates or products of the same reaction). A metabolite gene-set is defined by the group of genes that are associated with reactions in which the metabolite participates in.

### Metabolic changes associated with lung adenocarcinoma transformation

To illustrate the benefits of exploiting the gene-set interaction network, compared to only considering the gene overlap, we re-analysed a differential gene expression dataset from lung adenocarcinoma vs. normal lung tissue [15]. Metabolites from the human genome-scale metabolic model HMR2 [16] were used as gene-sets (i.e. genes associated with reactions in which a specific metabolite participates) and the GSA was carried out using the Bioconductor R-package piano [1], which produces files that can be directly imported by Kiwi. The Kiwi network (Figure 2a) clearly identifies significant gene-sets composing two metabolically connected pathways. For example, 5-phosphoribosylamine and 1-pyrroline-5-carboxylate both participate in pyrimidine biosynthesis, but their relatedness becomes apparent if the underlying metabolic network that measures the mutual distance is considered. These important connections are lost when the results are presented as a traditional heatmap (Figure 2b) or a network based on overlap of gene members of the different gene-sets (Figure 2c). The Kiwi heatmap (Figure 2d) shows the gene-level transcriptional changes for each gene-set enabling the identification of interacting gene-sets without gene overlap, and their driver-genes. For example, 5-phosphoribosylamine is a significant gene-set because of *GART* and *PPAT* up-regulation, while 1-pyrroline-5-carboxylate is significant due to *LEFTY1* and *PYCR* up-regulation. The heatmap also simplifies the detection of similar gene-sets, as e.g. nLc6Cer[c] and paragloboside[c].

### Metabolic changes associated with activation of oncogenic Kras in mouse tumor xenografts

Using a second case study we sought to test if Kiwi is able to reproduce networks known to be informative in a certain condition. To this end, we re-analyzed gene expression data from a study where the oncoprotein Kras was conditionally activated in mouse xenograft tumors [17]. The authors showed that activation of oncogenic Kras entails extensive metabolic reprogramming, in particular up-regulation of steroid biosynthesis. We therefore performed GSA [1] in the context of a mouse genome-scale metabolic network (Figure 3a) and tested if Kiwi could capture the relevant network of gene-sets upon Kras activation. In line with the results in the aforementioned study, we observe the emergence of the steroid biosynthetic pathway, which is overexpressed in different steps (Figure 3b). Indeed, despite the fact that isopentenyl-pPP, 14-demethyllanosterol, squalene, and lanosterol are not overlapping gene-sets (as shown by the heatmap in Figure 3c), Kiwi relates the metabolites given their vicinity in the underlying mouse metabolic network. Notably, contrary to the gene-set enrichment analysis used by the authors, Kiwi also identifies which pathway among the different branches of steroid biosynthesis is truly up-regulated by Kras activation, namely lanosterol synthesis.

## Conclusions

Kiwi is a new tool tailored for the visualization of GSA results in a gene-set interaction network context. As opposed to available tools, Kiwi starts from the premise that gene-sets can be precise biological entities that achieve a certain function by means of their interactions, such as metabolites in a pathway. This paradigm significantly improves the interpretation of the effect of transcriptional regulation in a certain context, such as metabolism, because it adds an extra layer of information to the GSA results. As exemplified in the two case studies, such addition is fundamental to capture certain transcriptionally regulated processes. In the case of the transformation to lung adenocarcinoma, we observe that the up-regulation of pyrimidine biosynthesis is mediated by the connection provided by choloyl-CoA. In the case of oncogenic Kras activation in mouse tumors, not only do we reproduce the up-regulation of the steroid biosynthetic process, but we also report that this is ascribed mainly to the synthesis of lanosterol. In neither case could such results be highlighted by connecting gene-sets using gene overlap (see Figure 2c) or by overlaying the GSA results on the corresponding gene-set interaction network (see Figure 3a). In favour of a clean layout for enhanced interpretation, Kiwi reduces the gene-set interaction network while maintaining and highlighting the important gene-set connections. It works with the output from any GSA tool and any collection of gene-sets that can be described as a network. For full usability, from raw data to final figure, it integrates seamlessly with the Bioconductor R-package piano (for GSA) and Cytoscape (for advanced layout and customization). Kiwi is available as a Python package at http://www.sysbio.se/kiwi and an online tool in the BioMet Toolbox at http://www.biomet-toolbox.org [19].

## Availability and requirements

**Project name:** Kiwi

**Project home page:**www.sysbio.se/kiwi

**Operating system(s):** Platform independent

**Programming language:** Python

**Other requirements:** Kiwi depends on the following python packages: numpy > = 1.8.0; matplotlib > = 1.3.1; networkx > = 1.8.1; mygene > = 2.1.0; pandas > = 0.13.1; scipy > = 0.13.3.

**License:** MIT

**Any restrictions to use by non-academics:** None

## Additional file

Additional file 1:
**Case study demo files.**
